# Inappropriate vitamin D supplementation among multimorbid older patients: a multicountry analysis

**DOI:** 10.1186/s12877-025-06189-w

**Published:** 2025-07-19

**Authors:** Elisavet Moutzouri, Shanthi Beglinger, Martin Feller, Anne Eichenberger, Olivia Dalleur, Wilma Knol, Marielle Emmelot-Vonk, Denis O’Mahony, Benoit Boland, Carole E. Aubert, Patricia O. Chocano-Bedoya, Drahomir Aujesky, Anne Spinewine, Nicolas Rodondi

**Affiliations:** 1 Institute of Primary Health Care (BIHAM), University of Bern, Bern, Switzerland; 2 Department of General Internal Medicine, Inselspital, Bern University Hospital, University of Bern, Bern, Switzerland; 3 Institute of Hospital Pharmacy, Bern University Hospital, University of Bern, Bern, Switzerland; 4 Louvain Drug Research Institute, Clinical Pharmacy and Pharmacoepidemiology research group, Université catholique de Louvain, Brussels, Belgium; 5 Pharmacy, Cliniques Universitaires Saint-Luc, Brussels, Belgium; 6 University Medical Center Utrecht, Utrecht University, Utrecht, Netherlands; 7 Department of Medicine, University College Cork & Department of Geriatric & Stroke Medicine, Cork University Hospital, Wilton, Cork, Ireland; 8 Geriatric, Medicine Division, Cliniques Universitaires Saint-Luc, Brussels, Belgium; 9 Institute of Health and Society, Université Catholique de Louvain, Belgium Louvain-la-Neuve,; 10 CHU UCL Namur, Pharmacy department, Yvoir, Belgium

**Keywords:** Vitamin D, Inappropriate prescribing, Overuse, Older adults

## Abstract

**Background:**

The prevalence of vitamin D supplementation and the percentage of participants with a lack of appropriate vitamin D supplementation (“potential underuse”) or potentially inappropriate vitamin D supplementation (“potential overuse”) and risk factors for these are currently unclear.

**Methods:**

Cross-sectional analysis from the OPERAM study, a multicenter cluster randomized controlled trial in four European countries (Belgium, Ireland, The Netherlands, Switzerland) including multimorbid (≥ 3 chronic conditions) older patients, with polypharmacy (≥ 5 chronic medications). For the definition of potential underuse and overuse we used high-risk conditions, which were defined according to the START criteria (version 2) for potential prescribing omissions in older people i.e., E2) long-term systemic corticosteroid therapy, known osteoporosis or osteopenia, E3) previous fragility fractures, and E5) housebound/in nursing homes or experiencing falls. We used mixed effect logistic regression to identify factors associated with underuse and overuse.

**Results:**

2008 patients (79.4y, SD 6.3, 45% female) were included. 825/2008 (41.1%) were supplemented with vitamin D. We identified 681 participants with potential underuse (33.9% of all participants, 69.7% of non-vitamin D users) and 204 with potential overuse (10.2% of all participants, 24.7% of vitamin D users). In the multivariable logistic regression analysis increasing age and being male were associated with underuse, while the number of baseline medications and previous hospitalizations were associated with both underuse and overuse. Specifically, underuse decreased with an increasing number of medications (OR: 0.93, 95% CI: 0.90–0.95), while overuse increased (OR: 1.08, 95% CI: 1.04–1.12). Previous hospitalizations were linked to underuse (OR: 1.08, 95% CI: 1.00-1.17) and inversely associated with overuse (OR: 0.88, 95% CI: 0.77–0.99).

**Conclusions:**

One-third of multimorbid older adults experienced potential underuse, while up to 10% potential overuse of vitamin D supplementation. Polypharmacy, previous hospitalizations, increasing age and being male are factors associated with inappropriate use of vitamin D. A better targeted vitamin D supplementation is warranted.

**Trial registration:**

NCT02986425, Registration date 2016-10-21.

## Key Points

**Question:**

Among older multimorbid patients, what are the rates of inappropriate vitamin D supplementation and risk factors of it?

**Findings:**

In this cross-sectional analysis of 2008 multimorbid older patients with polypharmacy, 41% received vitamin D supplementation, of which 10% did not belong to high-risk groups, representing potential overuse. A further 34% of all participants were not receiving vitamin D, although belonging to high-risk groups, i.e. representing potential underuse. In multivariable analyses specific risk factors for underuse or overuse of vitamin D were identified: polypharmacy, hospitalizations, age and sex.

**Meaning:**

These findings show considerable vitamin D treatment variations with evidence of inappropriate use in multimorbid older people. The uncertain data regarding the clinical impacts of vitamin D may be the reason for these results. A better targeted prescription of vitamin D supplementation is warranted.

## Introduction

Hypocalcemia or osteomalacia resulting from vitamin D deficiency is currently uncommon in most industrialized countries [1]. Nonetheless, low vitamin D levels are highly prevalent, though definitions and thresholds remain debated, and currently, no widespread agreement exists on “normal values” of 25-hydroxy-vitamin-D (25-OH-D) [2–4].

**Table 1:** Baseline characteristics of included participants

Variable	All participants (*n* = 2008)	Vitamin D users (*n* = 825, 41.1%)	Non-Vitamin D users (*n* = 1183, 55.9%)	*P*-value
Age, y (mean, SD)	79.4 (6.3)	80.0 (6.4)	78.9 (6.2)	0.000
Female (n, %)	898 (44.7)	471 (57.1)	427 (36.1)	0.000
BMI kg/m^2^ (mean, SD) ^a^	27.0 (5.7)	26.5 (6.22)	27.2 (5.4)	0.004
History of weight loss ^a^	694 (34.6)	303 (36.7)	391 (33.0)	0.094
Obesity	687 (34.2)	276 (33.5)	411 (34.7)	0.550
Smoking^b^(n, %)				
Yes	158 (7.9)	60 (7.3)	98 (8.3)	0.399
Alcohol (n, %)^c^				
No	1185 (59)	635 (56.6)	669 (56.6)	0.002
History of fractures (n, %)	259 (12.9)	145 (17.6)	114 (9.6)	0.000
Osteoporosis (defined as T-Score<−2.5 SD)	226 (11.3)	181 (21.9)	45 (3.8)	0.000
Corticosteroid use^d^	261 (13.0)	158 (19.2)	103 (8.7)	0.000
Calcium supplementation (n, %)	179 (8.9)	177 (22)	2 (0.2)	0.000
Polypharmacy				
Number of drugs (mean, SD)	10.5 (4.5)	12.5 (4.9)	9.1 (3.5)	0.000
≥7 drugs (N,%)	1,659 (82.6)	772 (93.6)	887 (74.9)	0.000
≥10 drugs (N,%)	1,032 (51.4)	574 (69.6)	458 (38.7)	0.000
No previous hospitalization in the last 6 months^e^	977 (48.7)	385 (46.7)	592 (50.0)	0.030
History of falls^f^	835 (41.6)	378 (45.8)	457 (38.6)	0.001
Is the patient housebound or living in institution (n, %)^g^	436 (21.7)	194 (23.5)	242 (20.5)	0.102
PCP visit in the last 6 months^h^ (mean, SD)	5 (5.1)	5.3 (5.2)	4.9 (5.0)	0.129
0–3 (n, %)	877 (47.6)	343 (46.0)	507 (48.9)	
4–6 (n, %)	669 (36.4)	264 (35.4)	405 (37.0)	
> 7 (n, %)	294 (16.0)	139 (18.6)	155 (14.2)	
Ethnicity: African (n, %)^i^	16 (0.8)	5 (0.6)	11 (0.9)	0.344
Education (n, %)^j^				
Less than high school	595 (30)	250 (30.3)	345 (29.2)	0.000
High School	909 (45)	417 (50.6)	492 (41.6)	0.000
University	475 (24)	150 (18.2)	325 (27.5)	0.000
Informed consent by Proxy (n, %)^k^	157 (7.8)	85 (10.3)	72 (6.1)	0.001
Chronic obstructive pulmonary disease (n, %)	376 (18.7)	165 (20.0)	211 (17.8)	0.221
Chronic kidney disease (n, %)	553 (27.5)	248 (30.1)	305 (25.8)	0.035
Medication adherence^l^	7 (6–7)	7 (6–7)	7 (6–7)	0.147

^a^BMI: *N* = 163 missing, history of weight loss: *N* = 32 missing, history of weight loss was collected through patient self-report at baseline, where participants were asked if they have lost weight within the past 12 month.^b^10 missing ^c^17 missing ^d^the exact time of corticosteroid use at hospital admission could not be retrospectively determined ^e^12 missing, defined in the table as current use vs. no, based on patient self-report at baseline (in the multivariable model units of alcohol/week) ^f^19 missing, information on falls was collected through patient self-report at baseline, where participants were asked about falls occurring within the past 12 months. A positive history was defined as having experienced more than one fall during that timeframe or a corresponding ICD code (see Appendix). ^g^8 missing, any time spent in a nursing home during the last 6 months ^h^168 missing, i6 missing, less than high school education (= 9 years of schooling completed), high school degree (high school completed), post-secondary degree (diploma from a university or an equivalent institution ^j^29 missing ^k^the variable serves as a surrogate marker for cognitive dysfunction ldetermined with the MMAS8 score (Morisky medication adherence measure questionnaire) Values ranged from 0 to 8. Higher scores indicate higher levels of adherence. Use of the ©MMAS is protected by US copyright laws. Permission for use is required. A license agreement is available from: Donald E. Morisky, ScD, ScM, MSPH, Professor, Department of Community Health Sciences, UCLA Fielding School of Public Health, 650 Charles E. Young Drive South, Los Angeles, CA 90095 − 1772, dmorisky@ucla.edu

**Table 2:** Underuse and overuse: cross-sectional analyses

Variable	All centers Number of participants (*N* = 2008) (%)	Bern Switzerland (*N* = 822)	Cork, Republic of Ireland (*N* = 346)	Louvain, Belgium (*N* = 388)	Utrecht, The Netherlands (*N* = 452)
Underuse					
No intake while indicated due to high-risk condition	681 (33.9%)	250 (30.4%)	132 (38.2%)	181 (46.7%)	118 (26.1%)
Overuse					
Intake with no high-risk condition	204 (10.2%)	106 (12.9%)	23 (6.7%)	23 (5.9%)	52 (11.5%)

High-risk conditions defined according to START E2, E3, and D3: patients taking long-term systemic corticosteroid therapy, patients with known osteoporosis or osteopenia, people with previous fragility fractures and older people housebound and in nursing homes or experiencing falls. No formal statistical testing was conducted for cross-country comparisons

**Table 3:** Multivariable adjusted odds ratios for factors related to underuse or overuse

Variables included in the model	Underuse		Overuse	
	OR (95% CI)	*P*-value	OR (95% CI)	*P*-value
Age (years)	1.02* 1.00-1.04	0.026	0.98 0.95–1.01	0.258
Sex (male vs. female)	1.24 0.99–1.54	0.054	0.87 0.63–1.22	0.440
BMI (kg/m2)	1.02 0.99–1.03	0.184	0.99 0.97–1.03	0.712
Smoking (yes/no)	1.23 0.83–1.85	0.289	0.75 0.4–1.40	0.384
Primary care physicians visits (number last 6 months)	1.01 0.99–1.03	0.083	0.98 0.96–1.02	0.496
Alcohol consumption (alcohol units per week)	0.98 0.97-1.00	0.116	1.00 0.98–1.03	0.795
Education Less than high school/high school vs. University	1.11 0.84–1.46	0.473	1.03 0.67–1.58	0.881
Number of baseline comorbidities	1.01 0.99–1.03	0.182	0.98 0.95-1.00	0.147
Number of baseline medications	0.93* 0.90–0.95	0.000	1.08* 1.04–1.12	0.000
Number of previous hospitalizations (last 6 months)	1.08* 1.00-1.17	0.029	0.88* 0.77–0.99	0.043
Informed consent by proxy (surrogate for cognitive decline)	1.04 0.70–1.58	0.858	0.44 0.18–1.05	0.063

*P < 0.05; non-linear variables (e.g. number of comorbidities, alcohol/week, number of drugs, primary care physician visits, number of previous hospitalizations) were explored using splines

According to one systematic review, 37.3% of studies worldwide reported an average 25-OH-D values < 50 nmol/l, showing a mild to moderate deficiency [5], depending on reference ranges for vitamin D [6]. Regarding older adults, the prevalence of vitamin D deficiency (defined as 25-OH-D < 50 nmol/l) in the United States between 2001 and 2006 for the population aged > 70 years old was 24% in males and 27% females [7]. In the SENECA study, an investigation of the diet and health of 824 older people from 19 towns in 11 countries (Greece, Portugal, Italy, Spain, France, Switzerland, Hungary, Belgium, Netherlands, Denmark and Norway) 36% of men and 47% of women had 25-OH-D concentrations below 30 nmol/l [8]. This is important considering differences in diet, lifestyle and sun exposure between countries. However, the value of population based vitamin D screening has not been proven and is not universally recommended, especially considering the exponential increase in vitamin D testing and associated costs in recent years [9]. 

Several studies from basic science to observational cohorts have highlighted an association between low vitamin D and chronic diseases, as well as acute conditions [7, 10–12]. However, large-scale randomized controlled trials (RCTs) with up to 25,874 patients and a follow-up of up to 5 years have shown no significant effects on several outcomes, including cardiovascular disease, diabetes, fractures, falls, cancer incidence and overall mortality [13–16]. The discrepancy between observational studies, which often show associations, and RCTs, which report no significant benefits, may be due to several factors. These include differences in study populations, such as the limited inclusion of severely deficient individuals in RCTs, and possible bias e.g. other co-interventions, which may confound the results [17]. Considerable debate continues as to the true benefits of vitamin D supplementation, especially in populations with severe vitamin D deficiency, since these patients were not adequately represented in RCTs [16]. Nevertheless, during the last decade several guidelines for screening and vitamin D supplementation have been generated, showing substantial variation in their recommendations. For example, both the US Preventive Services Task Force and the recently published guideline from the Endocrine Society advise against routine screening for vitamin D deficiency. However, while the US Preventive Services Task Force recommends supplementation only for individuals with low vitamin D- without specifying particular risk groups– the Endocrine Society recommends a supplementation for several at-risk populations, such as individuals aged 75 years or older and people with prediabetes [18, 19]. 

To better understand the public health opportunities for vitamin D supplementation, current patterns of vitamin D supplementation need to be assessed especially in specific populations at risk of deficiency, such as older patients and also people with multimorbidity, who are often not considered in clinical guidelines or in trials but represent a growing population [20]. Our study aims to find out how many older multimorbid patients are treated or potentially lack treatment with vitamin D, and for which corresponding indication and potential explanations. The results of this study may guide further research on screening criteria and targeted supplementation strategies.

## Methods

### Objectives

1. To identify current prevalence of vitamin D supplementation in older hospitalized multimorbid adults in four European countries.
2. To identify the percentage of multimorbid older patients with a lack of appropriate vitamin D supplementation (“potential underuse”) and potentially inappropriate vitamin D supplementation (“potential overuse”)..
3. To determine which factors are associated with “potential underuse” and “potential overuse”..

### OPERAM trial study design, setting and participants

We used cross-sectional data of the Optimizing Therapy to Prevent Avoidable Hospital Admissions in Multimorbid Older Adults (OPERAM): OPERAM trial, a two-arm, cluster-randomized controlled trial on a population of 2008 multimorbid patients (experiencing three or more chronic conditions concurrently) with associated polypharmacy (defined as five or more different regular drugs), who were ≥ 70 years old, recruited on hospital wards of four countries (Belgium, Ireland, The Netherlands, Switzerland). This European Horizon 2020 funded project explored the impact of a software-based decision medication review system based on STOPP/START version 2 criteria versus usual care on rehospitalization rates [21, 22]. Patients were recruited from hospital wards such as geriatrics, internal medicine, and surgery, with common diagnoses including cardiovascular diseases, diabetes, osteoporosis, fractures and others. Exclusion criteria were: planned transfer to palliative care within 24 h after admission, report of any structured drug review within two months before enrolment, and the inability to provide written informed consent or to obtain written informed consent from a proxy.

In OPERAM, all diagnoses, as well as all medications, were documented for each participant at hospital admission and recorded by using international ICD − 10 diagnostic codes and ATC medication codes by trained medical doctors and pharmacists, in a uniform manner across all centers. For the current project, we identified data for vitamin D users and non-users at the point of their admission in the hospital for all participants (Tables 4 and 5 in Appendix). We further identified the vitamin D dose in all users. Of note vitamin D use was assessed based on all medications reported at baseline, using a combination of prescription data and patient-reported use, and coded using ATC classifications. This approach allowed us to capture both prescribed and over-the-counter (OTC) vitamin D products, including those taken as standalone supplements or as part of multivitamin preparations. This study was not a pre-specified analysis, however vitamin D use was documented as part of the OPERAM design, which prespecified documentation of all current medications.

### Outcomes

The high-risk conditions were defined as persons identified by START (E2, E3, E5) criteria used in the OPERAM study using ICD-10 and ATC codes, as well as baseline questionnaires (Tables 4 and 5 in Appendix). START E2, E3, E5 criteria includes the following: E2) patients taking long-term systemic corticosteroid therapy, patients with known osteoporosis or osteopenia (bone mineral density (BMD) −1 to −2.5/<−2.5, respectively, measured with dual energy x-ray absorptiometry (DEXA)), E3) patients with previous fragility fractures and E5) older people who are housebound or in nursing homes or experiencing falls. STOPP/START criteria for potentially inappropriate prescribing in older people are criteria validated by an international European panel of experts in geriatric pharmacotherapy [21]. 

Potential underuse was defined by the presence of a corresponding indication (i.e. high-risk condition, as defined above) without vitamin D supplementation; potential overuse if vitamin D was prescribed without a high-risk condition.

Since universal screening of vitamin D levels are not recommended, we did not use vitamin D levels to define our high-risk groups. However, for the subgroup of patients from Switzerland (*N* = 822), the largest recruiting center, we applied an additional definition accounting for 25-OH-D levels. Specifically, we defined potential overuse as no high-risk condition and 25-OH-D level ≥ 50 nmol/l, and potential underuse as a high-risk condition and 25-OH-D levels < 50 nmol/l. A blinded study nurse collected 25-OH-D measurements up to one year before OPERAM study enrollment by contacting the primary care physicians (PCPs) of the patients or looking through the hospital records.

Some guidelines like for example the European society for clinical and economic aspects of osteoporosis and osteoarthritis (ESCEO) [23] or the American Geriatrics Society [24] recommend vitamin D screening in high-risk groups defined as bone diseases, old age, dark skin, obesity, sports, chronic kidney disease, liver failure, malabsorption syndromes, medication (antiepileptic drugs, glucocorticoids, AIDS drugs, antifungals, cholestyramine) and granulomaforming diseases. For this reason, we decided to make a sensitivity analyses using the groups for which data was available: patients with chronic kidney disease, liver failure, malabsorption syndromes, medications (antiepileptic drugs, glucocorticoids, AIDS drugs, antifungals, cholestyramine) or obesity, in addition to the groups defined from START2 criteria (Appendix Table 7) [23]. Some specific subgroups like dark skin were not included in this definition given the specifics of our population (up to 99% Caucasian). In addition, factors like sun-exposure and diet were not assessed in this cohort.

### Statistical analysis

We used descriptive statistics to describe the baseline characteristics of the included participants, stratified according to vitamin D supplementation and the potential underuse and overuse of vitamin D. Baseline characteristics were compared using the chi-squared test for categorical variables and the t-test for continuous variables, if the distribution was normal and non-parametric tests such as the Mann-Whitney U test for continuous variables without a normal distribution.

Initially, following a scoping review and expert opinion we examined the association between potential underuse and overuse with the following factors: age, sex, body mass index (BMI), alcohol consumption (self reported, units/week), education (less than high school education (= 9 years of schooling completed), high school degree (high school completed), post-secondary degree (diploma from a university or an equivalent institution)) and number of PCP visits in the previous 6 months [25]. In a second step, considering our specific population and statistical significant differences at baseline, we added following parameters in the models: number of comorbidities at baseline, number of baseline drugs, number of previous hospitalizations during the last 6 months, “informed consent by proxy”, as a proxy for cognitive impairment in the model. Variables that were used to define underuse or overuse (e.g. osteoporosis) were not included in the model. We used multivariable logistic mixed-effect regression models including country as a random effect variable in the model. The models for potential underuse and potential overuse were run separately, each using multivariable logistic regression. We explored non-linear relationships using splines in the following categories: multimorbidity (6, 11, 22 number of comorbidities) and in polypharmacy (6, 10, 17 number of medications). We analyzed age subgroups: 70–75, 75–80 and > 80. Results for each factor are reported as odds ratios (OR) with 95% confidence intervals (CI).

All analyses were performed using STATA (version 16, College Station, TX).

## Results

### Patients’ characteristics

Patients were recruited between 1 December 2016 and 31 October 2018. A total of 2008 patients (898 [44.7%] female sex) were included in our study, among which 825 (41.1%) were prescribed vitamin D. Mean (SD) age was 79.4 years (6.3). Mean (SD) BMI was 27 kg/m2 (5.7). The majority of our population was Caucasian (self-report) and only 16/2008 (0.8%) were non-Caucasian (Table 1).

The percentage of smokers in our study population was low 158/2008 (7.9%). Compared to vitamin D non-users, vitamin D users had more fractures or history of osteoporosis (17.6% vs. 9.6%) and more falls (45.8% vs.38.6%). Further, a history of a previous hospitalization in the previous 6 months was more frequent in vitamin D-users as compared to non-vitamin D-users. 436/2008 (21.7%) of patients were housebound or living in institution (any time spent in a nursing home during the last 6 months) (self-reported) and this proportion was equally distributed among vitamin D- users vs. non-users. Almost half of our study population had a PCP-visit in the last 6 months, with similar numbers among vitamin-D users vs. non-users. In the non-users group, there were more patients with a university education. Among participating centers, vitamin D use was less in Louvain (Belgium), which also had a lower prevalence of osteoporosis, a finding that could be explained by a slightly younger population compared to the other centers (mean age 78.5 years, SD 5.9) (Appendix Table 6) or differences in fortification of foods with vitamin D of health policies [26]. 

### Underuse and overuse

By using START criteria, we identified 681 persons with potential underuse (33.9% of all participants, 69.7% of vitamin D non-users) and 204 with potential overuse (10.2% of all participants, 24.7% of vitamin D-users) (Table 2).

Among the 204 participants with potential overuse, 98 (52%) had a dose of 800 unit/day. There were 2 persons receiving 300,000 units and 600,000 units/week, 1 person was receiving 7500 units/day, and 1 person was receiving 25,000 units/day. The rest were receiving doses under 4000 Units/day, a level the Institute of Medicine recommends not exiding [3]. However, no person among those exiding the recommended doses reported adverse effects.

Underuse was similar for the Bern and Louvain centers, more in the Cork center and less in the Utrecht center. Overuse was slightly more prevalent in Bern and Utrecht (Table 2).

In the initial model, only being male was significantly associated with vitamin D underuse (OR 1.31, 95%CI 1.06–1.62). However, after adding the number of comorbidities, baseline medications, previous hospitalizations and informed consent by proxy (factors that differed significantly between groups at baseline), sex was only marginally significant (*p* = 0.05) while a significant association between the number of baseline medications and both underuse and overuse was found: underuse decreased with an increasing number of medications (for each additional medication OR: 0.93, 95% CI: 0.90–0.95), while overuse increased (OR: 1.08, 95% CI: 1.04–1.12). Further, previous hospitalizations were linked to underuse (for each additional hospitalization OR: 1.08, 95% CI: 1.00-1.17) and inversely associated with overuse (OR: 0.88, 95% CI: 0.77–0.99). Increasing age was associated with underuse (Table 3). Sensitivity analyses by age groups stratifications did not show a particular trend.

Among participants in Switzerland, 186/822 (23%) of participants with under or overuse patients had their levels of 25-OH-D measured within one year before study enrollment. Taking into account 25-OH-D levels to define potential underuse and overuse, we identified 26.8% patients with potential underuse (30.4% without taking into account 25-OH-D levels) and 10.2% with potential overuse (12.9% without taking into account 25-OH-D levels).

Had we taken into account further “high-risk groups” i.e. kidney disease, liver disease, malabsorption syndromes, medications affecting vitamin D metabolism, adipositas the percentage of underuse increases to 50% of multimorbid adults.

Further sensitivity analysis by removing this corticosteroid use (*N* = 261) in the definition of overuse and underuse (since the exact time of corticosteroid use could not be retrospectively determined) showed similar results with our main definition (Appendix Table 5).

## Discussion

In the present cross-sectional analysis of 2008 multimorbid patients in four large European medical centers, we found that 41.1% of the population were vitamin D users. We identified 10% patients with potential overuse, and 33.9% with potential underuse.

In our cohort, notable results included a significant association between the number of baseline medications and both underuse and overuse: underuse decreased with an increasing number of medications, while overuse increased. Polypharmacy may represent more intensive medical management, where vitamin D is prescribed routinely or preemptively, potentially leading to overuse but at the same time reducing underuse. In this case medication reviews may help address this issue. Additionally, previous hospitalizations were linked to underuse and inversely associated with overuse. Participants who were hospitalized in the previous 6 months were more likely to underuse and less likely to overuse vitamin D. A possible explanation could be the loss of patient care continuity following frequent hospitalizations, which underscores the need for continuity of care in multimorbid older people. Further specific reasons may include the potential role of discharge prescription changes or other patient related factors. In this direction, implementing care models involving regular follow-ups to the general practitioner may be important and should be further evaluated [27]. 

A further factor which was marginally related with vitamin D underuse was being male. Most studies e.g. for osteoporosis treatment have been performed in women and therefore considerable uncertainty remains in treating older men, which possibly explains the underuse of vitamin D in men [28]. An association between PCP- visits and vitamin D underuse or overuse was not identified. Neither did we find any significant association with BMI, smoking or alcohol consumption. OPERAM patients had a very high level of medication adherence as measured by the Medication Adherence Questionnaire (©MMAS-8) [29, 30], which includes questions on discontinuing medications without guidance of the physician which renders non-adherence to prescribed medications less likely as the reason for underuse. Drug adherence was not different between vitamin-D users and non-users. Regarding cross-country differences, we noted small differences with underuse being more prevalent in Belgium and Ireland, while overuse was higher in Switzerland. Healthcare system variations, like over-the-counter medications (OTC) or other factors like vitamin D fortifications policies or different guidelines between the countries could be possible explanations for the differences, underscoring the importance of considering intercountry differences in international or European guidelines. A further aspect could be that the cost of screening for 25-OH-D is higher than the cost of vitamin D supplementation, which may contribute to the overuse of supplementation, especially in settings where screening is not routinely conducted. However, these factors were not explored in the present cohort.

Increasing age was associated with vitamin D underuse. Older people may experience decreased endogenous vitamin D synthesis due to possible limited outdoor sunlight exposure and age-related decline in production of vitamin D through UV in the skin [31]. However, several clinical studies have failed to provide compelling evidence of supplementing vitamin D at a population level for several outcomes, although there are no studies that focus specifically on multimorbid older people [24, 32–36]. According to the most recently published guidelines of the US Preventive Services Task Force, evidence to recommend supplementing all older adults with vitamin D to prevent fractures or other diseases is not currently available [37]. However, these guidelines do not apply in high-risk groups, which may benefit from vitamin D supplementation [9, 38]. We identified that a considerable number of multimorbid older patients belonging to high-risk groups received no vitamin D supplementation. Though it is important to avoid overscreening, it is important not to miss persons belonging to these high-risk groups. The underuse of vitamin D among older adults may be attributed to several factors, among them, the lack of awareness about high-risk conditions, a desire to minimize polypharmacy, unclear or conflicting guidelines, and controversies surrounding the perceived benefits of vitamin D supplementation and further research is needed. The inclusion of further high-risk groups, as described from several guidelines [23, 24] in the high-risk definition in our cohort of multimorbid patients resulted in a strikingly high proportion of underuse. This is particularly important in the context of multimorbid older individuals. This should be taken into account when considering guidelines for screening and supplementation, since this could lead to overscreening particularly in this population. Specific studies and guidelines for multimorbid individuals are needed.

Among individuals flagged for potential vitamin D overuse in the present cohort, more than half (52%) were taking the standard preventive dose of 800 IU/day. Only a small number received very high doses, including two individuals on 300,000 and 600,000 IU/week, respectively and two others on 7,500 and 25,000 IU/day. No adverse effects were reported among those exceeding the recommended doses. However, current evidence warns against intermittent high-dose regimens, such as monthly boluses, which have been linked to increased fall risk, particularly in older adults. Specifically, two large RCTs—the VIDA trial (100,000 IU/month) and D-Health trial (60,000 IU/month)—showed no musculoskeletal benefit and raised safety concerns [16, 18]. Extremely high doses like 300,000–600,000 IU/week are not supported in clinical guidelines and pose a risk of toxicity.

The strengths of this study include the multicenter approach of the study, involving four large European centers, increasing the representativeness and generalization of the results and the inclusion of 2008 multimorbid patients, which provides valuable insights into an often overlooked population. The assessment of medication adherence using the Medication Adherence Questionnaire (©MMAS-8) adds an important dimension to the study, helping to rule out non-adherence as a potential cause for vitamin D underuse. Further strengths include the accurate documentation of medications using ATC codes, including over-the-counter (OTC) medications, the accurate description of comorbidities using ICD-10 codes, as well as including patients through informed consent by proxy, a group commonly excluded from studies, which further strengthens the application of our findings in this special geriatric patient group.

This study also has some limitations. Firstly, serum vitamin D measurements were not available in three out of four participating centers. As such, the definitions of “potential underuse” and “potential overuse” rely exclusively on the START criteria and does not incorporate serum 25-OH-D levels for most participants. However, universal screening is currently not recommended and thus our classification is guideline-based and do not incorporate biochemical verification of vitamin D status (25-OH-D levels) for most participants. We partly addressed this limitation by performing a sensitivity analysis for the site center of Bern, which included 822 patients, accounting for 25-OH-D levels and showed that the results remained similar. However, the possibility of misclassification cannot be excluded. Second, while the START criteria provided a standardized approach to identifying prescribing appropriateness, they are not specific to vitamin D under or overuse and may not capture all relevant clinical nuances. We did not systematically assess cognitive status, caregiver involvement, or frailty, all of which may influence prescribing decisions. While “informed consent by proxy” served as a proxy for decisional impairment, and if a patient was housebound or living in an institution was assessed, these do not capture the full complexity of these factors. This may have contributed to unmeasured confounding in our analysis of underuse and overuse. A further limitation is the lack of details about the alimentary sources of vitamin D or sun exposure in this patient cohort. However, alimentation represents only about 10% of vitamin D source; further, only a few foods contain vitamin D, i.e. vitamin D3, naturally in quantities that have an impact on the dietary intake: fish liver, fish liver oils, and fatty fish and egg yolks. In addition, DEXA measurements were missing in a large proportion of participants and therefore some patients with osteopenia or osteoporosis as a clear indication for vitamin D supplementation may not have been detected. However, we would have expected that if a DEXA measurement had shown osteoporosis the diagnosis would have been detected as an ICD code. The study was conducted in four European countries, which may limit the generalizability of the results to other populations and cultural contexts. These findings should be examined in further multicounty studies. Vitamin D use was assessed based on all medications reported at baseline, using a combination of prescription data and patient-reported use, this approach allowed us to capture both prescribed and over-the-counter (OTC) vitamin D products. However, we acknowledge that reliance on self-report and recorded medication lists may still result in some underreporting, particularly for non-prescription supplements. Further, in the present analysis we addressed the prevalence of vitamin D use with a cross-sectional methodology but did not assess the clinical impacts of underuse or overuse. However, cohort studies are not sufficient to establish a cause-and-effect relationship. Lastly, the cross-sectional design of our study precludes causal inferences and may not fully capture temporal relationships between risk factors and prescribing behaviors.

## Conclusion

In this first multicenter study providing real-life high-quality data from a population often excluded from clinical studies, one third of older adults experienced potential underuse in regards to vitamin D, while up to 10% potential overuse. Our data show that health policy makers may have to meet further measures to promote a more adequate use of vitamin D. Further research, including improved international guidelines for vitamin D prescribing, clearer high-risk group definitions and better integration of multimorbidity factors into recommendations are needed. Further it is crucial to conduct secondary analyses of already published trials e.g. by using individual participant data, relevant to high-risk groups like multimorbid adults and adults with specific chronic diseases like CKD or liver disease as well as patients with very low 25-OH-D levels and analyses regarding dosage recommendations.

## Data Availability

Access to the datasets may be granted for research purposes, subject to institutional and ethical approvals where applicable.

## Appendix

**Table 4:** Definitions for vitamin D & calcium using ATC codes

Vitamin D*	A11CC01-06, A11CC20
Vitamin D combinations with Calcium or biphosphonates or Vitamin A^a^	A11CC, A12AX, A11CB, A11CC55, M05BB03-09
Multivitamins with minerals	A11AA03
Calcium (and combinations)	A12AA, A12A, A12AA20, A11AA02, A11GB01, A11JB, A11EC, A12AX

^a^included in the definition of “vitamin D users”

**Table 5:** ICD and ATC codes used for definitions of START E2,E3,E5

Osteoporosis	M80*, M81*, + bmd where available
Fractures	S12*,S22*,S32*,S42*,S52*,S62*,S72*,S82*,S92*,T02*,T08, T10, T12, T14.2
Osteoporosis medication	M05BA01-8, M05BB01-8, M05BX4-6
Corticosteroid use	H02AB01, H02AB02, H02AB04, H02AB06, H02AB07, H02AB09, H02AB13
Falls & housebound	R29.6, R26.3, W01, W05, W06, W07, W08, W10, W18, W19 + baseline questionnaires (at least one fall last year and/or if the patient is bedridden or independent)

**Table 6:** ICD and ATC codes for expanded group of high-risk groups

COPD	J44*
Obesity	E66*
Chronic kidney disease	N19, N18, N18.1-5, N18.8-9, N18.80, N18.89, N25.0
Mineral metabolic disorders (Hypophosphatemia, rickets)	E83.38, E83.30, E83.31, E83.3, E.83.39
Malabsorption through different reasons	K52.8, K52.9, K52.3, K52.30, K 52.31, K 32, K38, K51, K50, K90*,K91.2, A09.9, Q43.8, K90.0, K83.2, K90.49, K90.4
Vitamin D deficiency	E55, E55.9, E55.0, E64.3
Hyperparathyreodism (primary)	E21.0-5
Liverscarring/liver Cirrhoris	K72.0, K72.40, K72.10, K72.48, K72.9, K72.18, K70.4, K70.41, K72, K72.1, K71.1, K70.42, K74.69, K70.30, K74.60, K71.70, K70.31, K76.1, K76, K74.5, K74.6, P78.81, K74.3
HIV medications	J05AR01-27
Antiepileptics	N03AB-G, NO3AX
Aromatase Inhibitors	L02BG01
Androgen	L02BB01-06
Colestyramine	C10AC01
Antimycotics for systemic use	J02AB01-2; J02AC01-06; J02AC01-2

**Table 7:** Variables according to center

	Overall *N* = 2008	Bern, Switzerlan *N* = 822	Cork, Republic of Ireland (*N* = 346)	Louvain, Belgium *N* = 388	Ultrecht, the Netherlands *N* = 452
Vitamin D use (n,%)	825 41.09	404 49.15	122 35.26	88 22.68	211 46.68
Osteoporosis (n, %)	226 11.25	108 13.14	41 11.85	14 3.61	63 13.94
Fractures (ICD) (n,%)	259 12.90	120 14.60	50 14.45	41 10.57	48 10.62
Falls_all (ICD + Questionnaires)	835 41.58	403 49.03	140 40.46	121 31.19	171 37.83
Housebound & beiing in an institution	436 21.71	117 14.23	94 27.17	436 21.71	84 18.58
Corticosteroid (n,%)	261/2008 (13%)	108/822 (13.1%)	48/346 (13.9%)	41/388 (10.6%)	64/452 (14.2%)

*19 missing

**Table 8:** Underuse and overuse excluding corticosteroids from the definitions

Variable	All centers Number of participants (%)
Underuse	
No intake while indicated due to high-risk condition	638 (31.7%)
Overuse	
Intake with no high-risk condition	257 (12.8%)

## Abbreviations

25-OH-D: 25-hydroxy-vitamin-D
BMD: Bone mineral density
BMI: Body mass Index
DEXA: Dual energy x-ray absorptiometry
©MMAS-8: Medication Adherence Questionnaire
OPERAM: Optimizing Therapy to Prevent Avoidable Hospital Admissions in Multimorbid Older Adults
PCPs: Primary care physicians
UV: Ultraviolet
